# Reply to Marciano and Rossiter: Balancing muscle oxygen consumption sampling and slope quality in NIRS‐derived assessment of muscle oxidative capacity

**DOI:** 10.1113/EP094495

**Published:** 2026-11-12

**Authors:** Guillaume Costalat, Benoît Sautillet, Grégoire P. Millet, Clément Unal, Abd‐Elbasset Abaïdia, Abdellah Hassar, Maryne Cozette

**Affiliations:** ^1^ Faculty of Sport Sciences, APERE laboratory, UR‐UPJV 3300 University of Picardie Jules Verne Amiens France; ^2^ Institute of Sport Sciences University of Lausanne Lausanne Switzerland; ^3^ Department of Kinesiology Laval University Quebec Canada

**Keywords:** methodology, muscle oxidative capacity, near‐infrared spectroscopy

1

Dear editor,

We thank Marciano and Rossiter (2026) for their thoughtful comments on our recent study (Costalat et al., 2026) and for highlighting important methodological considerations regarding the assessment of near‐infrared spectroscopy (NIRS)‐derived muscle oxidative capacity. We are pleased that our work contributes to the ongoing discussion on how to improve the measurement and reliability of this increasingly used technique. We agree that further methodological work is important and welcome the opportunity to discuss some of the points raised in their letter.

Marciano and Rossiter appropriately emphasize that the early, steep portion of the muscle oxygen consumption ($m{\dot{V}}_{O_{2}}$) recovery curve contains particularly valuable information compared with the later asymptotic portion. Based on this rationale, the authors suggest that increasing the frequency of $m{\dot{V}}_{O_{2}}$ measurements during this early phase, by shortening the occlusion–reoxygenation cycles, might allow a better characterization of the steep portion of the recovery curve, thereby improving the estimation of NIRS‐derived muscle oxidative capacity (i.e., the rate constant, *k*). In our view, this approach might introduce several potential limitations that we believe are important to highlight.

Increasing the sampling frequency of $m{\dot{V}}_{O_{2}}$ raises possible technical considerations. In our preliminary testing, shortening the reoxygenation intervals between successive occlusions compromised the ability of our rapid cuff‐inflation system (E20/AG101, Hokanson) repeatedly to reach the target pressure rapidly and consistently. We also caution authors and readers against interpreting our findings as evidence that 3 s (or shorter) arterial occlusions are sufficient. In our study, 3 s slopes were extracted from 6 s occlusions. Our results therefore support the use of a shorter analytical window within a longer occlusion (Costalat et al., 2026) but do not establish that a 3 s occlusion itself would yield an equivalent measurement. More specifically, a 3 s occlusion cannot be assumed to provide 3 s of valid deoxygenation data, because part of this interval is already taken up by cuff inflation and the time needed to reach the target pressure, and signal artefacts can also occur around cuff inflation and deflation. Moreover, as occlusion duration decreases, these technical constraints will, inevitably, affect an increasingly larger proportion of the available measurement window. Taken together, these considerations highlight the risk of incomplete arterial occlusion, which might alter the deoxygenation slope and thus affect the estimated *k*.

Marciano and Rossiter also advocate the use of a saturation‐clamp approach (Pilotto et al., 2022; Villanova et al., 2025), in which the exercise stimulus is adjusted such that occlusions can begin without delay after exercise, allowing higher‐frequency sampling of the early $m{\dot{V}}_{O_{2}}$ recovery while constraining tissue oxygenation within a narrow range. Although this is an attractive concept, the extent to which this approach improves the robustness of *k* compared with conventional time‐based protocols remains to be firmly established. We acknowledge that the saturation‐clamp approach standardizes local oxygenation, an important determinant of the measured *k*. However, this might come at the cost of greater variability in the preceding exercise stimulus. Specifically, because tissue saturation reflects the dynamic balance between O_2_ utilization and O_2_ delivery (Grassi & Quaresima, 2016), achieving the same target saturation might require tailoring the exercise stimulus between individuals, thereby introducing differences in exercise intensity and/or duration. Importantly, Tripp et al. (2024) demonstrated that contraction intensity per se affects NIRS‐derived muscle oxidative capacity during voluntary exercise. They reported a substantially greater time constant (τ) following contractions at 100% of maximal voluntary contraction than at 40% (34.4 vs. 24.2 s), corresponding to *k* values of 1.82 and 2.68 min^−1^, respectively. The authors suggested that differences in muscle fibre recruitment contributed to this effect and, consequently, emphasized the importance of standardizing contraction intensity. Although achieving a given target tissue saturation is unlikely to require differences in contraction intensity as large as those examined by Tripp et al. (2024), smaller inter‐individual differences might still influence the estimated *k* through variability in the exercise stimulus preceding recovery. Importantly, this effect might be specific to voluntary contractions, because Ryan et al. (2013) reported no influence of contraction intensity when using electrical stimulation, which recruits muscle fibres in a different manner from voluntary contractions. Therefore, in our view, this point deserves particular attention given the widespread use of voluntary exercise in contemporary NIRS‐derived muscle oxidative capacity protocols (Beever et al., 2020; Zuccarelli et al., 2020).

A further practical consideration is the potential operator dependence of the saturation‐clamp approach. The maintenance of tissue saturation within a narrow range requires continuous adjustment of occlusion and release durations based on real‐time monitoring of the NIRS signal, potentially increasing the influence of operator experience and decision‐making. This could, in turn, contribute to measurement variability. Thus, intra‐ and inter‐operator reliability should be established before improved reliability is claimed as an advantage of this approach.

Finally, although we acknowledge that the time‐based approach (6 s occlusion–10 s release) used in our study provides fewer measurements of $m{\dot{V}}_{O_{2}}$ during the steep portion of recovery, this limitation can be offset, in part, by the rigorous quality‐control applied to each individual deoxygenation slope. In our study, slope selection was fully standardized and automated, with only monotonic 3 s segments displaying an *R* ^2^ > 0.99 being retained. This highly restrictive criterion was applied specifically to exclude poorly fitted or artefact‐contaminated slopes and minimize operator influence. Conversely, in the saturation‐clamp approach first introduced by Pilotto et al. (2022) and advocated by Marciano and Rossiter, criteria for accepting or rejecting individual deoxygenation slopes were not reported explicitly. Therefore, although this approach inherently increases the number of $m{\dot{V}}_{O_{2}}$ measurements during early recovery, the inclusion of slopes with inconsistent goodness of fit cannot yet be excluded. In other words, when estimating *k*, more is not necessarily better; greater $m{\dot{V}}_{O_{2}}$ sampling frequency should not come at the expense of individual slope quality.

In summary, rather than favouring either approach, we advocate direct comparisons of time‐based and saturation‐clamp protocols in future methodological studies. The key question is whether the benefits of greater $m{\dot{V}}_{O_{2}}$ sampling frequency and tighter control of tissue oxygenation outweigh the potential variability associated with individualized exercise stimuli, very short occlusion windows (3 s or shorter) and real‐time protocol adjustment. The most robust approach might not be the one that provides the greatest number of $m{\dot{V}}_{O_{2}}$ measurements, but the one that yields the most valid and reliable measurement of NIRS‐derived muscle oxidative capacity.

## AUTHOR CONTRIBUTIONS

All authors have approved the final version of the manuscript and agree to be accountable for all aspects of the work in ensuring that questions related to the accuracy or integrity of any part of the work are appropriately investigated and resolved. All persons designated as authors qualify for authorship, and all those who qualify for authorship are listed.

## CONFLICT OF INTEREST

The authors declare no conflicts of interest.

## GENERATIVE AI STATEMENT

Not used.
